# MCID, SCB, and PASS Thresholds After Percutaneous Trigger Finger Release With or Without Corticosteroid Injection

**DOI:** 10.3390/jcm15166299

**Published:** 2026-08-14

**Authors:** Recep Karasu, Mustafa Dinç, Gökay Eken

**Affiliations:** 1 Department of Orthopedics and Traumatology, Bursa City Hospital, Bursa 16250, Turkey; drindianster@gmail.com; 2 Hand Surgery Division, Department of Orthopaedics and Traumatology, Faculty of Medicine, Bursa Uludağ University, Bursa 16059, Turkey; gokay_eken@yahoo.com

**Keywords:** trigger finger, percutaneous release, patient-reported outcome measures, minimal clinically important difference, substantial clinical benefit, patient acceptable symptom state, Michigan Hand Outcomes Questionnaire, corticosteroid

## Abstract

**Background/Objectives**: Percutaneous A1 pulley release effectively treats trigger finger, but the additional benefit of concomitant corticosteroid injection remains debated. Moreover, minimal clinically important difference (MCID), substantial clinical benefit (SCB), and patient acceptable symptom state (PASS) thresholds have not been well established for this procedure, limiting patient-centered interpretation of outcomes. **Methods**: This retrospective cohort study included 80 patients (40 per group) who underwent percutaneous trigger finger release alone (PR) or with adjunctive triamcinolone injection (PR+CS). Anchor-based MCID and SCB thresholds for QuickDASH (Quick Disabilities of the Arm, Shoulder and Hand), Michigan Hand Outcomes Questionnaire (MHQ), and visual analog scale (VAS) pain were derived using GROC as the external anchor, whereas PASS thresholds were derived using a dichotomous acceptable-state question; ROC analysis was used for all threshold estimates. Group comparisons incorporated effect sizes and responder analyses. **Results**: MCID thresholds were ΔVAS ≥ 3, ΔQuickDASH ≥ 19, and ΔMHQ ≥ 16.5; SCB thresholds were ΔVAS ≥ 4, ΔQuickDASH ≥ 25, and ΔMHQ ≥ 20; and PASS thresholds were 3-month VAS ≤ 4, QuickDASH ≤ 29, and MHQ ≥ 76. For MCID and SCB, MHQ showed the highest observed AUCs (0.899 and 0.946, respectively). Greater mean improvements in all change scores were observed in the PR+CS group than in the PR group (ΔQuickDASH 24.2 vs. 19.9, *p* < 0.001, d = 1.39; ΔMHQ 20.5 vs. 15.0, *p* < 0.001, d = 1.67). However, the magnitudes of the between-group differences did not reach the derived MCID or SCB thresholds. PASS achievement was numerically higher in the PR+CS group than in the PR group (75% vs. 55%), but this difference did not reach statistical significance (*p* = 0.061). **Conclusions**: Preliminary MCID, SCB, and PASS estimates were derived for percutaneous trigger finger release; the principal novelty lies in the procedure-specific SCB and PASS estimates, with the SCB estimates being, to our knowledge, the first reported for any trigger finger treatment modality. MHQ showed the highest observed AUCs for MCID and SCB, whereas QuickDASH showed the highest observed AUC for PASS, underscoring the complementary information provided by the three instruments. Although greater mean functional changes were observed in the PR+CS group, the magnitudes of the between-group differences did not reach the derived MCID or SCB thresholds. However, given the retrospective, non-randomized design, these between-group findings remain exploratory and hypothesis-generating and should be viewed as a patient-centered re-interpretation of an established clinical question rather than as new comparative evidence; no causal benefit of adjunctive corticosteroid injection can be inferred. Given the small anchor-defined derivation subgroups and the potential instability of the selected cut-offs, these candidate estimates require prospective external validation in independent cohorts before clinical application.

## 1. Introduction

Trigger finger (stenosing tenosynovitis) is a common hand disorder characterized by painful clicking, locking, or catching of the affected digit during flexion and extension. It is estimated to affect approximately 2–3% of the general population [1,2]. The underlying pathophysiology involves a mismatch between the flexor tendon and its surrounding sheath at the level of the A1 pulley, leading to fibrocartilaginous thickening, increased friction, and impaired tendon gliding [3,4]. These mechanical and inflammatory changes result in pain, functional limitation, and reduced hand performance, substantially impacting daily activities and work-related tasks.

Percutaneous release of the A1 pulley has become a widely accepted minimally invasive surgical option for the treatment of trigger finger. Compared with open release, percutaneous techniques are associated with shorter operative times, minimal soft tissue disruption, faster recovery, and high success rates when performed by experienced surgeons [5,6,7,8].

However, trigger finger pathophysiology involves not only mechanical stenosis of the A1 pulley but also a distinct inflammatory component—namely tenosynovitis and synovial hypertrophy—which may persist even after successful mechanical decompression [9,10]. Therefore, addressing the mechanical obstruction alone may be insufficient to optimize early postoperative recovery [1]. To target this persistent inflammatory component, some authors have advocated adjunctive corticosteroid injection at the time of percutaneous release to enhance early functional recovery [11,12,13]. Although the existing literature includes comparative studies and evidence syntheses, differences across outcome domains and follow-up intervals complicate patient-centered interpretation of the reported effects.

Traditionally, outcomes following trigger finger surgery have been evaluated using statistical comparisons of patient-reported outcome measures (PROMs), such as the Quick Disabilities of the Arm, Shoulder and Hand (QuickDASH) questionnaire or hand-specific instruments like the Michigan Hand Outcomes Questionnaire (MHQ) [14,15]. These measures provide complementary perspectives on postoperative recovery: QuickDASH assesses region-specific upper-extremity symptoms and disability, MHQ provides a hand-specific multidimensional assessment, and VAS isolates pain intensity as a distinct symptom domain. Their concurrent use allows functional recovery and pain relief—related but distinct components of trigger finger outcomes—to be evaluated within the same cohort. While these tools are validated and widely used, interpretation of their results has largely focused on statistical significance rather than clinical relevance [16]. Statistical significance alone does not necessarily indicate that an observed change is meaningful to patients, particularly in conditions such as trigger finger where baseline disability may be moderate and postoperative improvement is often rapid and substantial [17,18,19,20,21].

In this context, patient-centered outcome thresholds—specifically the Minimal Clinically Important Difference (MCID), Substantial Clinical Benefit (SCB), and Patient Acceptable Symptom State (PASS)—have emerged as essential concepts for interpreting treatment success [20,22,23]. MCID represents the smallest change in an outcome measure that patients perceive as beneficial, SCB defines a higher threshold of change associated with substantial perceived benefit, and PASS defines the absolute symptom level beyond which patients consider their condition acceptable or satisfactory. Together, these thresholds provide a clinically interpretable framework that complements traditional statistical analyses and is increasingly recommended for outcome evaluation in musculoskeletal research [20,24,25]. Despite their growing importance, MCID, SCB, and PASS values have not been systematically established for percutaneous trigger finger release, limiting the ability to contextualize postoperative improvements and compare results across studies.

Although MHQ MIC/MCID values have previously been reported after open release and conservative management [17,18], to our knowledge, SCB and PASS estimates specific to percutaneous release have not been reported; in particular, SCB has not previously been defined for any trigger finger treatment modality. Accordingly, the objectives of this study were: (1) to derive preliminary procedure-specific estimates of MCID, SCB, and PASS for QuickDASH, MHQ, and VAS pain scores in patients undergoing percutaneous trigger finger release, using an anchor-based methodology with ROC curve analysis; (2) to apply these patient-centered metrics to the comparison of patient-reported outcomes between percutaneous release alone and release with adjunctive corticosteroid injection, thereby providing a patient-centered re-interpretation of an established clinical question rather than new comparative evidence.

## 2. Materials and Methods

### 2.1. Study Design and Ethical Approval

This study was conducted as a single-center, retrospective cohort study evaluating patient-centered outcomes following percutaneous trigger finger release with or without adjunctive corticosteroid injection. Consecutive patients treated at the Department of Orthopedics and Traumatology, Bursa City Hospital, Bursa, Türkiye, between January 2022 and December 2023 were retrospectively reviewed. No a priori power analysis or formal sample-size calculation was performed. The sample comprised all eligible consecutive patients treated during the study period who met the inclusion criteria and had complete required outcome data. Therefore, the final cohort size was determined by case availability rather than statistical considerations and should be regarded as a consecutive convenience sample. The study protocol was approved by the Bursa Uludağ University Faculty of Medicine Health Research Ethics Committee (Approval No. 2025/927/18-18; Date: 22 October 2025). All procedures were conducted in accordance with the ethical principles of the Declaration of Helsinki and its subsequent amendments. Due to the retrospective design of the study and the use of anonymized clinical data, the requirement for individual informed consent was waived by the Institutional Review Board. The study involved secondary analysis of pre-existing clinical data recorded during routine care before ethics approval; no research-specific patient contact or prospective data collection was undertaken.

### 2.2. Patient Selection

Patients were eligible for inclusion if they were between 18 and 75 years of age, had a clinical diagnosis of idiopathic trigger finger involving a single digit, and presented with Green grade 2 to 4 severity. Grade 1 (mild) cases were excluded because they typically respond well to conservative management and rarely require surgical intervention. Additional inclusion criteria included symptom duration exceeding three months despite conservative management, failure of at least one prior corticosteroid injection or patient preference for surgical treatment after informed discussion, availability of complete baseline patient-reported outcome measures, and ability to complete validated Turkish-language questionnaires.

Exclusion criteria comprised inflammatory or systemic rheumatologic disease (such as rheumatoid arthritis or psoriatic arthritis), previous surgery on the affected hand or digit, multiple-digit trigger finger requiring simultaneous surgical intervention, use of anticoagulant therapy that could not be safely paused for the procedure, known allergy to local anesthetics or corticosteroids, pregnancy or lactation, and cognitive impairment precluding reliable completion of questionnaires.

### 2.3. Group Allocation and Surgical Technique

Patients were categorized into two groups based on operative note documentation in the electronic health record: the percutaneous release (PR) group, comprising patients who underwent percutaneous trigger finger release alone, and the percutaneous release with corticosteroid injection (PR+CS) group, comprising patients who underwent percutaneous release followed by adjunctive corticosteroid injection. Adjunctive corticosteroid administration was not governed by a prespecified institutional protocol, formal scoring system, or standardized eligibility criteria. Instead, the decision was made by the treating surgeon on the basis of individual clinical judgment and practice preference at the time of the procedure. The specific patient-level rationale for corticosteroid administration was not consistently documented in the retrospective records and therefore could not be reliably reconstructed. Consequently, treatment allocation was non-random and may have been susceptible to selection bias and confounding by indication. All procedures were performed according to the institutional standard protocol by two senior hand surgeons, each with more than ten years of experience, to ensure procedural consistency. Surgeries were conducted under local anesthesia using 5 mL of 1% lidocaine without epinephrine, with application of a digital tourniquet.

The affected digit was identified clinically, and the A1 pulley was localized by palpation at the level of the distal palmar crease for fingers or the metacarpophalangeal joint flexion crease for the thumb. A 19-gauge needle was inserted perpendicular to the skin and advanced to the level of the A1 pulley, and release was performed using controlled transverse sweeping motions until intraoperative criteria for complete release were met. Complete release was defined as the absence of palpable catching or locking during full active flexion and extension of the digit performed by the patient upon verbal command, with smooth tendon gliding confirmed intraoperatively.

In the PR+CS group, 20 mg of triamcinolone acetonide (1 mL; Kenacort-A^®^, Bristol-Myers Squibb) was administered following confirmation of complete release. Correct intra-sheath placement was confirmed by initial resistance to injection followed by smooth flow of the solution and mild visible distension along the tendon sheath proximal to the A1 pulley. The needle was slightly withdrawn prior to corticosteroid administration to minimize the risk of intratendinous injection. In the PR group, the needle was withdrawn immediately after confirmation of complete release without injection.

### 2.4. Postoperative Management

Postoperative care was standardized for all patients. A small adhesive dressing was applied, and immediate unrestricted active range-of-motion exercises were encouraged. Patients were advised to resume daily activities as tolerated. Formal hand therapy was not routinely prescribed and was reserved for patients demonstrating persistent stiffness or limited motion beyond two weeks postoperatively.

### 2.5. Data Source and Outcome Measures

As part of the institution’s standard clinical pathway, patients routinely completed the Quick Disabilities of the Arm, Shoulder and Hand (QuickDASH) questionnaire, the Michigan Hand Outcomes Questionnaire (MHQ), and a visual analog scale (VAS) for pain electronically via tablet-based systems at each scheduled preoperative and postoperative visit.

The three routinely collected instruments were analyzed together because they assess complementary rather than interchangeable domains of recovery. The QuickDASH is an 11-item, region-specific measure of upper-extremity symptoms and disability, scored from 0 to 100, with lower scores indicating better function; its inclusion also facilitates comparison with the broader hand and upper-extremity literature [14]. The MHQ is a 37-item, hand-specific multidimensional instrument assessing overall hand function, activities of daily living, work performance, pain, aesthetics, and satisfaction, with higher scores indicating better outcomes [15]. The VAS provides a unidimensional assessment of pain intensity on a 0-to-10 scale, allowing pain-related recovery to be distinguished from broader functional recovery. Their concurrent use therefore enabled a multidimensional assessment encompassing regional upper-extremity disability, hand-specific function, and pain, while allowing comparison of their anchor-based discriminative performance within the same cohort. All patient-reported outcome data, together with demographic and clinical variables, were retrospectively extracted from the electronic health record.

### 2.6. Follow-Up and Anchor-Based Measures

Patient-reported outcome measures were assessed preoperatively and at 3 months postoperatively. At routine 3-month postoperative visits, the GROC and PASS anchor questions were administered as components of the department’s standard clinical follow-up protocol, independently of the present research, alongside the QuickDASH, MHQ, and VAS. Responses were recorded contemporaneously in the electronic health record before ethics approval. No additional patient contact was undertaken, and no GROC or PASS data were collected specifically for research purposes. Following ethics approval, the investigators retrospectively extracted these pre-existing clinical data. The 3-month time point was selected as the primary endpoint for threshold determination, consistent with the established window for early postoperative recovery following percutaneous trigger finger release [8,13].

At the 3-month follow-up, patients completed two anchor questions. The Global Rating of Change (GROC) was administered as a 5-point Likert scale: 1 = much worse, 2 = worse, 3 = unchanged, 4 = better, and 5 = much better [20]. The PASS anchor asked: “Considering your daily activities, work, hobbies, and pain, would you consider your current hand condition satisfactory or acceptable?” with responses recorded as “Yes” or “No” [24].

### 2.7. Determination of MCID, SCB, and PASS

MCID, SCB, and PASS estimates were derived for the overall study cohort (*n* = 80) using anchor-based methodology, independent of treatment group allocation. This pooled derivation was intended to generate preliminary procedure-specific estimates irrespective of corticosteroid administration; their generalizability beyond the present cohort requires external validation.

MCID was defined as the smallest difference a patient can perceive in a specific outcome measure, representing the minimum threshold of a noticeable and beneficial treatment effect [20]. SCB was defined as the magnitude of treatment-associated improvement that a patient recognises as a meaningful benefit, representing a higher threshold of recovery beyond merely noticeable change [23]. PASS was defined as the absolute value on a patient-reported outcome measure scale beyond which patients consider themselves well or in a satisfactory state, irrespective of the change from baseline [22].

The GROC scale served as the external anchor for MCID and SCB, whereas the dichotomous PASS question served as the anchor for PASS. Threshold derivation followed two steps. First, anchor responses were used to define positive and reference groups. For MCID, patients reporting a GROC score of 4 or 5 (“better” or “much better”; *n* = 29) formed the positive group, whereas those reporting a score of 1 or 2 (“much worse” or “worse”; *n* = 26) formed the reference group; patients reporting no change (GROC = 3; *n* = 25) were excluded from this analysis. For SCB, patients reporting GROC = 5 (“much better”; *n* = 25) formed the positive group, and all other patients (GROC ≤ 4; *n* = 55) formed the reference group. For PASS, patients answering “Yes” to the PASS question formed the positive group (*n* = 52), whereas those answering “No” formed the reference group (*n* = 28). Second, separate ROC analyses were used to identify the PROM change score that best distinguished the anchor-defined groups for MCID and SCB and the absolute 3-month PROM score that best distinguished the groups for PASS.

### 2.8. Statistical Analysis

All statistical analyses were performed using SPSS Statistics version 27.0 (IBM Corp., Armonk, NY, USA). The distribution of continuous variables was assessed using the Kolmogorov–Smirnov test, and the data were found to be predominantly normally distributed. Accordingly, continuous variables were presented as mean ± standard deviation (SD). Comparisons between the two independent groups were conducted using the independent samples *t*-test for continuous variables. Categorical variables were analyzed using the chi-square test or Fisher’s exact test, as appropriate. Effect sizes were calculated to complement *p*-values: Cohen’s d was used for continuous variables and Cramér’s V for categorical variables, interpreted as small, moderate, or large according to standard conventions.

To evaluate the construct validity of the GROC anchor, Spearman correlation analysis was performed between GROC scores and change (delta) scores for each outcome measure. For each outcome measure and threshold category, ROC curve analysis was performed and the area under the curve (AUC) with 95% confidence intervals (CI) was calculated to assess discriminative ability. Optimal cut-off values were determined using the Youden index (sensitivity + specificity − 1). In practical terms, the selected cut-off represented the score that provided the best balance between correctly identifying patients who met the relevant anchor criterion and correctly identifying those who did not. Sensitivity, specificity, positive predictive value (PPV), and negative predictive value (NPV) were reported for each optimal threshold. A *p*-value < 0.05 was considered statistically significant. Exploratory post hoc power and sample-size calculations were performed solely to contextualize the observed findings and were not used to determine the original cohort size. For PASS, calculations assumed a two-sided comparison of two independent proportions, equal group allocation, and α = 0.05, using the observed rates of 0.55 and 0.75. The sample size required for 80% power was estimated under the same assumptions. For the continuous change-score comparisons, post hoc power was contextualized from the observed Cohen’s d values under a two-sided independent-samples *t*-test framework.

## 3. Results

### 3.1. Anchor Validity

A strong and statistically significant correlation was observed between GROC scores and all change (delta) outcome measures, supporting the construct validity of the GROC scale as an external anchor. GROC demonstrated a very strong correlation with ΔMHQ (ρ = 0.859, *p* < 0.001), a strong correlation with ΔQuickDASH (ρ = 0.753, *p* < 0.001), and a moderate correlation with ΔVAS (ρ = 0.472, *p* < 0.001). Among the delta measures, moderate inter-correlations were observed between ΔMHQ and ΔQuickDASH (ρ = 0.432, *p* < 0.001) and between ΔMHQ and ΔVAS (ρ = 0.351, *p* = 0.001), whereas the correlation between ΔQuickDASH and ΔVAS was weak and did not reach statistical significance (ρ = 0.178, *p* = 0.114). These findings supported the suitability of GROC as an external anchor, with associations observed across functional and pain-related change scores.

### 3.2. ROC-Derived Thresholds for MCID, SCB, and PASS

**Figure 1:**
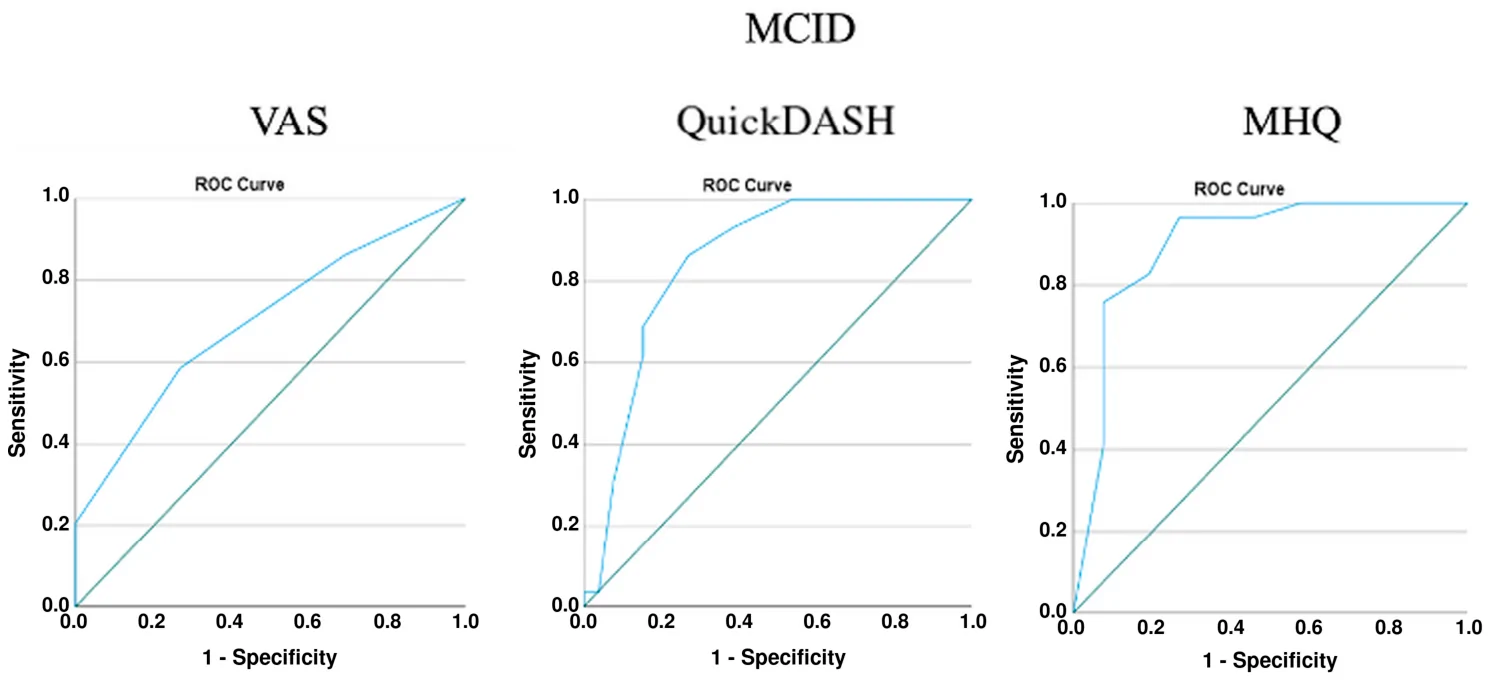
ROC curves for MCID determination at 3 months postoperatively. Curves are shown for ΔVAS (AUC: 0.700; **left**), ΔQuickDASH (AUC: 0.847; **center**), and ΔMHQ (AUC: 0.899; **right**). Optimal cut-off values were determined using the Youden index. The diagonal reference line represents chance-level discrimination (AUC = 0.50). MCID: minimal clinically important difference; AUC: area under the curve; VAS: visual analog scale; QuickDASH: Quick Disabilities of the Arm, Shoulder and Hand; MHQ: Michigan Hand Outcomes Questionnaire.

**Figure 2:**
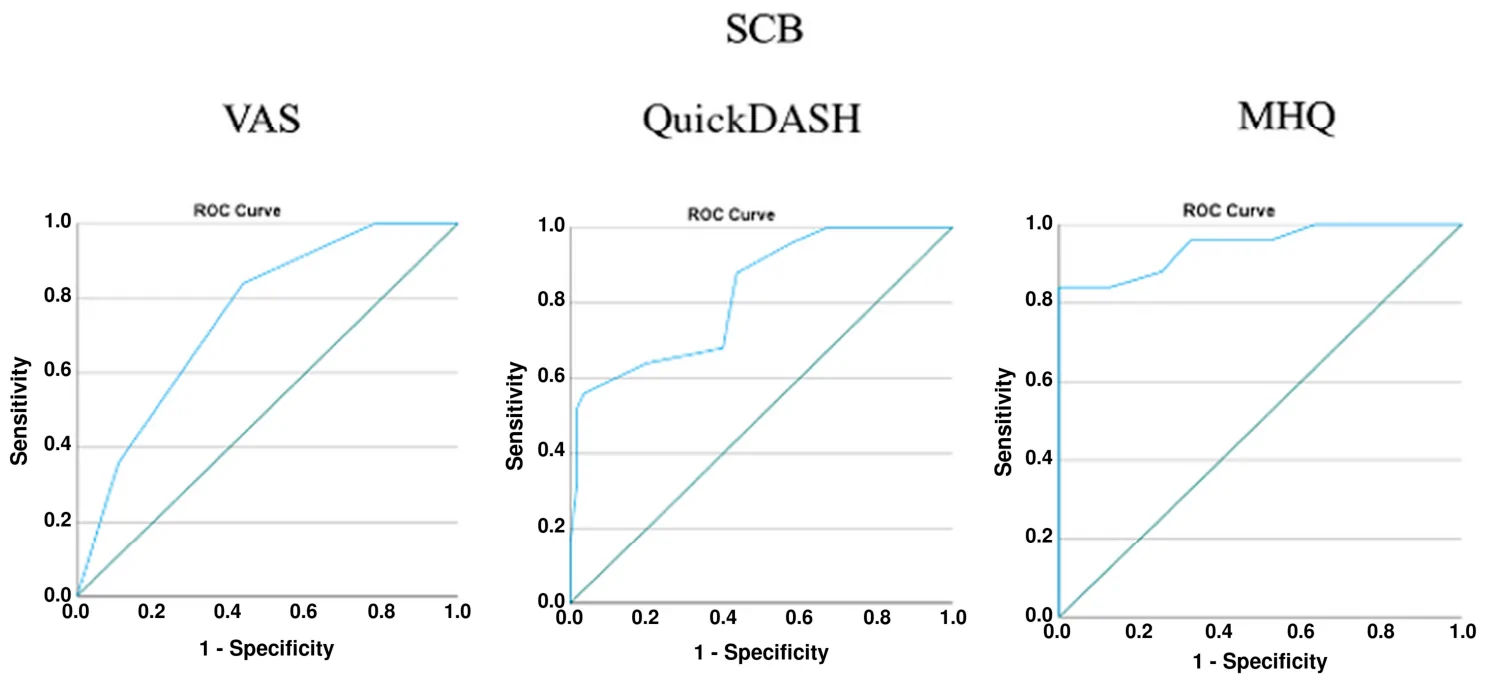
ROC curves for SCB determination at 3 months postoperatively. Curves are shown for ΔVAS (AUC: 0.752; **left**), ΔQuickDASH (AUC: 0.823; **center**), and ΔMHQ (AUC: 0.946; **right**). SCB: substantial clinical benefit; AUC: area under the curve.

**Figure 3:**
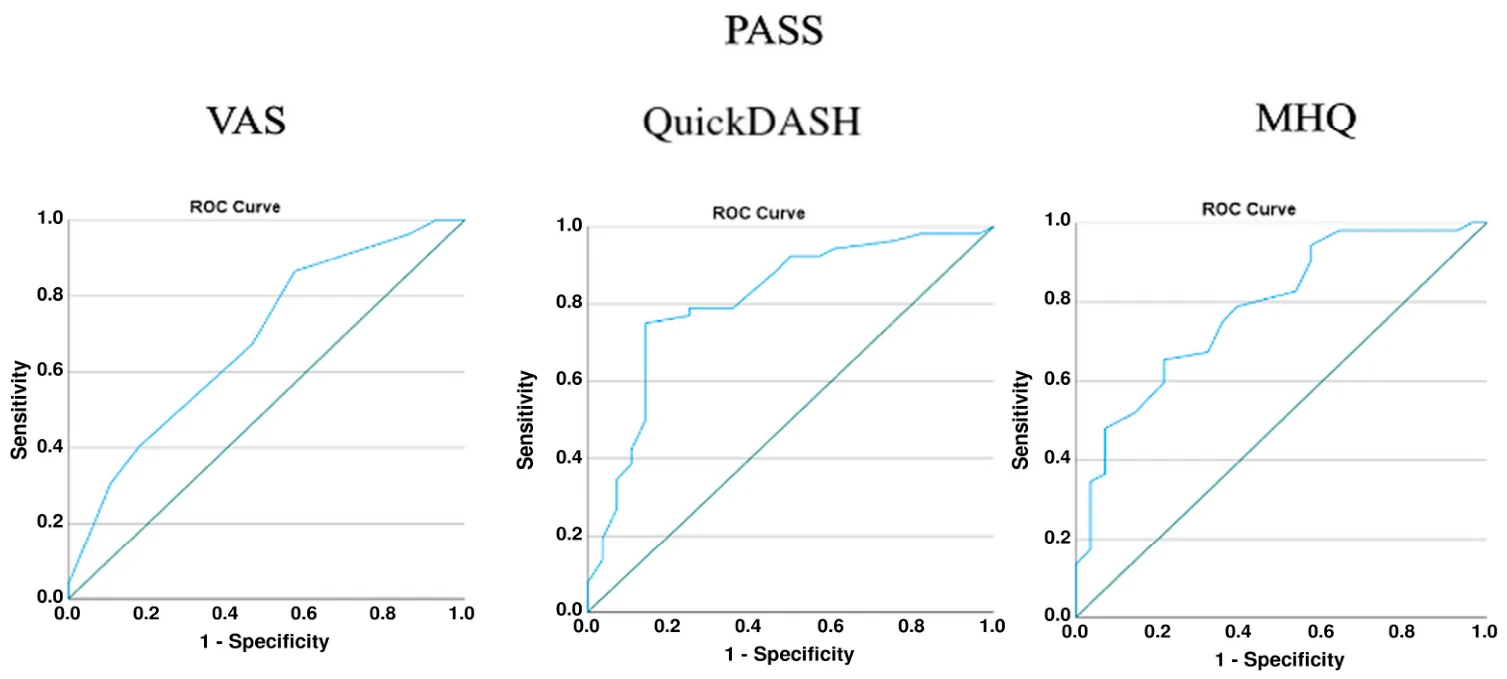
ROC curves for PASS determination at 3 months postoperatively. Curves are shown for month 3 VAS (AUC: 0.683; **left**), QuickDASH (AUC: 0.809; **center**), and MHQ (AUC: 0.784; **right**). For PASS analyses, absolute postoperative scores—rather than change scores—were used as the test variable. PASS: patient acceptable symptom state; AUC: area under the curve.

ROC analyses demonstrated varying degrees of discriminative ability across all outcome measures and threshold categories (Table 1; Figure 1, Figure 2 and Figure 3).

For MCID, the observed AUC was highest for ΔMHQ (0.899, 95% CI: 0.807–0.990), followed by ΔQuickDASH (0.847, 95% CI: 0.736–0.958) and ΔVAS (0.700, 95% CI: 0.562–0.837). The corresponding MCID cut-off values were ΔVAS ≥ 3, ΔQuickDASH ≥ 19, and ΔMHQ ≥ 16.5 points (Figure 1).

For SCB, the observed AUC was highest for ΔMHQ (0.946, 95% CI: 0.889–1.000), followed by ΔQuickDASH (0.823, 95% CI: 0.725–0.921) and ΔVAS (0.752, 95% CI: 0.644–0.860). The corresponding SCB cut-off values were ΔVAS ≥ 4, ΔQuickDASH ≥ 25, and ΔMHQ ≥ 20 points (Figure 2).

For PASS, the observed AUC was highest for the 3-month QuickDASH score (0.809, 95% CI: 0.706–0.913), followed by MHQ (0.784, 95% CI: 0.681–0.887) and VAS (0.683, 95% CI: 0.560–0.806). The corresponding PASS thresholds were 3-month VAS ≤ 4, QuickDASH ≤ 29, and MHQ ≥ 76 (Figure 3).

### 3.3. Baseline Demographic and Clinical Characteristics

A total of 80 patients were included in the analysis, with 40 patients in each group (PR and PR+CS). Baseline demographic and clinical characteristics were comparable between groups, with no statistically significant differences observed in age (52.1 ± 6.7 vs. 53.4 ± 6.4 years, *p* = 0.377), symptom duration (7.0 ± 1.9 vs. 6.8 ± 1.5 months, *p* = 0.563), baseline QuickDASH (48.5 ± 6.8 vs. 49.3 ± 7.2, *p* = 0.623), baseline MHQ (61.5 ± 7.5 vs. 60.7 ± 6.5, *p* = 0.623), and baseline VAS (6.5 ± 1.6 vs. 7.0 ± 1.4, *p* = 0.141). Sex distribution, diabetes mellitus prevalence, thumb involvement, and Green classification grade distribution were also similar between groups (all *p* > 0.05; Table 2).

### 3.4. Postoperative Outcomes at 3 Months

At 3 months postoperatively (Table 3), mean QuickDASH scores were lower in the PR+CS group than in the PR group (25.1 ± 7.3 vs. 28.6 ± 8.0, *p* = 0.044, d = 0.46), whereas mean MHQ scores were higher (81.2 ± 7.6 vs. 76.5 ± 8.4, *p* = 0.010, d = 0.59). Both comparisons yielded moderate effect sizes. Mean VAS scores were also lower in the PR+CS group (2.8 ± 1.6 vs. 3.5 ± 1.7), although this difference did not reach statistical significance (*p* = 0.063, d = 0.42).

Change-score analysis showed greater mean improvements in the PR+CS group for QuickDASH (24.2 ± 3.0 vs. 19.9 ± 3.2, *p* < 0.001, d = 1.39), MHQ (20.5 ± 3.3 vs. 15.0 ± 3.3, *p* < 0.001, d = 1.67), and VAS (4.2 ± 0.72 vs. 3.0 ± 0.78, *p* < 0.001, d = 1.59). PASS achievement was numerically higher in the PR+CS group (75.0% vs. 55.0%); however, the difference did not reach statistical significance (*p* = 0.061, V = 0.21), with a small-to-moderate effect size.

## 4. Discussion

The present study had two objectives. First, we derived preliminary ROC-based MCID, SCB, and PASS estimates specific to percutaneous trigger finger release. Because the anchor-defined derivation subgroups were small, the resulting cut-offs should be regarded as provisional and potentially unstable pending external validation. The principal novelty lies in the SCB and PASS estimates for this procedure; to our knowledge, the SCB estimates are the first reported for any trigger finger treatment modality. The MHQ MCID estimate should instead be interpreted as a percutaneous-release-specific extension of previously reported estimates from open release and conservative treatment. Second, we applied these patient-centered metrics to the established question of adjunctive corticosteroid use to provide a complementary interpretation of the comparative findings.

The significant correlations between GROC and the change scores supported the suitability of GROC as an external anchor. The comparative performance of the three instruments was domain-dependent. MHQ showed the highest observed AUCs for MCID and SCB, consistent with the ability of a hand-specific multidimensional instrument to capture functional change after trigger finger release. In contrast, QuickDASH showed the highest observed AUC for PASS, indicating that broader upper-extremity symptoms and disability may also contribute to whether patients consider their postoperative state acceptable. VAS provided a distinct pain-specific assessment but showed comparatively lower discrimination, suggesting that pain intensity alone may not fully represent patient-perceived recovery. Thus, these instruments should be viewed as complementary rather than interchangeable. Because formal responsiveness analyses and pairwise statistical comparisons of AUCs were not performed, the findings should be interpreted as differences in observed discriminative performance rather than evidence that any single instrument is universally superior or more responsive. The anchor-defined subgroups were small (MCID positive/reference: *n* = 29/26; SCB positive/reference: *n* = 25/55; PASS positive/reference: *n* = 52/28), limiting the precision and potential stability of the selected cut-offs. This uncertainty is reflected in several wide AUC confidence intervals; notably, the ΔVAS MCID AUC was 0.700 (95% CI, 0.562–0.837), with the lower bound approaching chance-level discrimination. Accordingly, all cut-offs should be interpreted as provisional derivation estimates rather than definitive clinical thresholds and require assessment in larger independent cohorts.

The high observed AUC (0.946) and particularly the 100% specificity of the candidate ΔMHQ SCB cut-off should be interpreted with caution, because perfect specificity in a small derivation subgroup may reflect derivation-sample optimism or overfitting. External validation is required before clinical application. By contrast, the MCID threshold for QuickDASH, while demonstrating good overall discrimination (AUC: 0.847), was associated with a relatively low specificity of 53.8%, indicating that approximately half of patients who did not achieve clinically meaningful improvement may nonetheless be classified as improved using this cut-off. This limitation should be considered when applying the ΔQuickDASH ≥ 19 threshold in clinical or research contexts. The comparatively modest performance of VAS for PASS may suggest that an acceptable postoperative state depends on dimensions beyond pain intensity alone, including broader functional recovery.

The comparative findings should be interpreted within a patient-centered framework. Greater mean improvements in QuickDASH and MHQ scores and large change-score effect sizes were observed in the PR+CS group; however, the non-randomized allocation precludes causal attribution. The between-group mean differences did not reach the candidate MCID or SCB thresholds. This discordance may occur in high-success procedures when both groups improve substantially, compressing the incremental difference below the level of individual perceptibility despite statistical significance. Accordingly, the findings do not confirm additional patient-perceived benefit and remain consistent with clinical equipoise.

The PASS analysis provides a complementary patient-centered perspective because it evaluates whether patients consider their postoperative condition acceptable or satisfactory, rather than the magnitude of change from baseline. PASS achievement was numerically higher in the PR+CS group than in the PR group (75.0% vs. 55.0%); however, the 20-percentage-point difference did not reach statistical significance (*p* = 0.061). This exploratory finding does not confirm a treatment effect and requires validation in adequately powered prospective studies.

Previous studies have reported clinically meaningful change thresholds for trigger finger in other treatment contexts. Koopman et al. [18] reported a triangulated MHQ MIC of 9.3 points after open release, whereas Atthakomol et al. [17] reported MCID values of 15 points for MHQ and 2 points for VAS pain after conservative treatment. Our anchor-based MHQ MCID estimate of 16.5 points for percutaneous release was numerically higher than the 9.3-point MIC reported after open release and close to the 15-point MCID reported after conservative treatment. Similarly, our VAS MCID estimate of 3 points was slightly higher than the 2-point value reported by Atthakomol et al. [17] after conservative treatment. However, differences in treatment context and derivation methodology, including the distinction between MIC and MCID, preclude direct equivalence among these estimates. The present MHQ MCID should therefore be interpreted as a percutaneous-release-specific estimate rather than as the first MHQ threshold reported for trigger finger. By contrast, the candidate SCB and PASS estimates appear to be the first reported specifically for percutaneous release, and, to our knowledge, the SCB estimates are the first reported for any trigger finger treatment modality. These candidate estimates remain preliminary and require external validation before broader clinical application. Contextualizing our thresholds against the broader hand surgery literature, Randall et al. [26] reported MCID values of 1.6–1.9 and SCB values of 2.2–2.6 for VAS pain in a postoperative hand and upper extremity population, while Hubbard et al. [27], in a heterogeneous nonshoulder hand surgery population, reported QuickDASH PASS thresholds of ≤15.9–20.5; our higher values likely reflect the greater baseline disability and expected recovery magnitude specific to surgical trigger finger populations.

Adjunctive corticosteroid injection during percutaneous release has already been evaluated in randomized trials and evidence syntheses that provide stronger comparative evidence than the present retrospective cohort, although findings vary across outcome domains and follow-up intervals. In a systematic review and meta-analysis encompassing over 4800 digits, Levine et al. [28] found no significant differences between percutaneous release alone and combined treatment in overall satisfaction, postoperative pain, recurrence, or need for open release, noting that the only significant QuickDASH difference was based on a single study with a small absolute effect. Similarly, Liu et al. [29] reported no significant improvement in return-to-activity or procedural success at 12 weeks with adjunctive corticosteroid use.

In contrast, multiple investigations have demonstrated early postoperative differences favoring the combined approach. In the only Level I randomized controlled trial on this topic (112 digits), Jegal et al. [13] reported significantly greater subjective improvement at 3 weeks with combined treatment, although differences diminished by 3 months—a temporal pattern consistent with the known pharmacological half-life of triamcinolone acetonide. Zan et al. [30] demonstrated significantly lower VAS scores and superior patient-reported improvement at 1 day and 1 month with ultrasound-guided combined treatment, with convergence at 1 year. Earlier investigations by Patel et al. [11], Ryu et al. [31] and Cebesoy et al. [32] consistently reported higher satisfaction rates and superior early symptom resolution with adjunctive corticosteroid use. The most recent systematic review by Wen et al. [12], encompassing 685 patients across seven studies, concluded that combined percutaneous release and corticosteroid injection positively affects patient-reported outcomes with a low complication rate of 8.8% and no major adverse events. Taken together, the Level I randomized trial by Jegal et al. [13] the large meta-analysis by Levine et al. [28] and the recent systematic review by Wen et al. [12] provide higher-level comparative evidence regarding the comparative effectiveness of adjunctive corticosteroid injection than the present retrospective cohort. Accordingly, our contribution is not new evidence of corticosteroid efficacy, but a patient-centered re-interpretation of this established question through the application of MCID, SCB, and PASS. This framework may inform future outcome interpretation and trial design, pending external validation of the derived thresholds.

Prior evidence suggests that adjunctive corticosteroid injection may accelerate early recovery, whereas longer-term between-group differences have not been consistently observed. This pattern is biologically plausible because trigger finger involves inflammatory tenosynovitis and synovial hypertrophy [3,4] and intra-sheath triamcinolone may suppress local inflammatory activity [33,34]. However, the present findings are only compatible with this mechanism; the non-randomized allocation and absence of objective inflammatory measurements preclude mechanistic or causal inference.

This study has several methodological strengths. MCID, SCB, and PASS estimates were derived from the overall percutaneous release cohort independently of treatment allocation, with the aim of generating preliminary procedure-specific rather than treatment-specific estimates. The observed correlations between GROC and the change scores supported the suitability of GROC as an external anchor; the 5-point GROC scale, exclusion of neutral responders, and dichotomous PASS anchor were consistent with the anchor-based analytical approach used in this study. The concurrent reporting of effect sizes alongside *p*-values provides a more complete characterization of between-group differences, and the simultaneous derivation of MCID, SCB, and PASS enables nuanced differentiation between minimal, meaningful, substantial, and satisfactory recovery levels.

First, the retrospective, non-randomized cohort design limits the strength of comparative inference between the PR and PR+CS groups and precludes causal conclusions. Because treatment allocation was based on intraoperative surgeon discretion rather than randomization, the observed between-group differences may have been influenced by unmeasured confounding, including surgeon-level practice patterns or patient-specific clinical and inflammatory characteristics that were not captured in the dataset. Although the groups were comparable with respect to the measured baseline characteristics, such comparability does not eliminate residual confounding or confounding by indication. Accordingly, the observed associations should not be interpreted as evidence of superiority or a definitive benefit of adjunctive corticosteroid injection; they remain exploratory and hypothesis-generating. The 3-month follow-up precludes assessment of long-term durability and recurrence, particularly relevant evidence from Jegal et al. [13] and Zan et al. [30] suggesting group differences may attenuate over time. Objective measures of tendon sheath inflammation were unavailable, and the proposed mechanistic framework therefore remains inferential. Regarding the derived thresholds, the 100% specificity observed for the MHQ SCB threshold warrants particular caution, as it is a potential indicator of overfitting to our specific derivation sample and may overstate the threshold’s diagnostic accuracy in broader clinical applications. These features underscore that all proposed cut-offs, particularly the MHQ SCB estimate, may be sample-dependent and require prospective external validation across diverse cohorts before broader clinical application. No a priori power analysis or formal sample-size calculation was performed, and the cohort size was determined by the availability of eligible consecutive patients with complete outcome data during the study period. An exploratory post hoc calculation based on the observed PASS rates (55% vs. 75%), 40 patients per group, and a two-sided α of 0.05 yielded approximately 47–50% power. Thus, a true 20-percentage-point difference had a relatively low probability of being detected in the present cohort. The observed *p*-value of 0.061 should neither be interpreted as confirmation of a treatment effect nor as evidence of equivalence, particularly because no equivalence margin or formal equivalence analysis was prespecified. Under the same assumptions, approximately 88–90 evaluable patients per group would be required to detect a 20-percentage-point difference with 80% power; allowing for modest attrition, a future trial could target approximately 100 patients per group. By contrast, the very large observed effect sizes for ΔQuickDASH (d = 1.39) and ΔMHQ (d = 1.67) corresponded to greater than 99% post hoc power under conventional two-sided independent-samples assumptions, suggesting that the principal power concern relates to the binary PASS outcome rather than these continuous change scores. These post hoc calculations should be interpreted as planning guidance for future research rather than as retrospective validation of the present findings. Finally, the single-center design and absence of formal inter-surgeon variability assessment limit generalizability.

Until externally validated, the proposed cut-offs should be used only as candidate research benchmarks rather than for individual clinical decision-making, with particular caution regarding the MHQ SCB and QuickDASH MCID estimates.

The comparative analysis neither supports routine adjunctive corticosteroid use nor excludes a clinically meaningful effect. Future randomized trials should be adequately powered for PASS and other prespecified patient-centered endpoints and should include longer follow-up and, where feasible, objective assessment of inflammatory burden.

## 5. Conclusions

This study derived preliminary procedure-specific MCID, SCB, and PASS estimates for percutaneous trigger finger release, with the candidate SCB and PASS estimates constituting the principal novel contribution. MHQ showed the highest observed AUCs for MCID and SCB, whereas QuickDASH showed the highest observed AUC for PASS. Given the small derivation subgroups, external validation is required before clinical adoption. Applied to the corticosteroid comparison, greater mean changes were observed in the PR+CS group, but the between-group differences did not reach the candidate MCID or SCB thresholds and the PASS difference was not statistically significant. These non-randomized findings provide a patient-centered re-interpretation rather than causal evidence of corticosteroid effectiveness and remain consistent with clinical equipoise. Confirmation requires adequately powered randomized trials.

## Figures and Tables

**Table 1:** ROC-Derived Thresholds and Diagnostic Performance for MCID, SCB, and PASS.

Parameter	MCID (VAS)	MCID (QuickDASH)	MCID (MHQ)	SCB (VAS)	SCB (QuickDASH)	SCB (MHQ)	PASS (VAS)	PASS (QuickDASH)	PASS (MHQ)
Positive (*n*)	29	29	29	25	25	25	52	52	52
Negative (*n*)	26	26	26	55	55	55	28	28	28
*p* value	0.011	<0.001	<0.001	<0.001	<0.001	<0.001	0.007	<0.001	<0.001
AUC	0.700	0.847	0.899	0.752	0.823	0.946	0.683	0.809	0.784
95% CI (AUC)	0.562–0.837	0.736–0.958	0.807–0.990	0.644–0.860	0.725–0.921	0.889–1.000	0.560–0.806	0.706–0.913	0.681–0.887
Sensitivity (%)	58.6	96.6	75.9	36.0	56.0	84.0	67.3	76.9	78.8
Specificity (%)	73.1	53.8	92.3	89.1	96.4	100	53.6	75.0	60.7
PPV (%)	71	66	92	53	87	100	72	85	79
NPV (%)	61	94	77	79	80	90	48	63	60
Threshold (cut-off)	≥3	≥19	≥16.5	≥4	≥25	≥20	≤4	≤29	≥76

MCID: minimal clinically important difference; SCB: substantial clinical benefit; PASS: patient acceptable symptom state; MHQ: Michigan Hand Outcomes Questionnaire; AUC: area under the curve; CI: confidence interval; PPV: positive predictive value; NPV: negative predictive value. Threshold values were determined using the Youden index (sensitivity + specificity − 1). For MCID and SCB analyses, higher delta values indicate greater clinical improvement, whereas for PASS analyses, higher MHQ and lower VAS and QuickDASH scores represent better clinical status. Sensitivity, specificity, PPV, and NPV are reported for the optimal cut-off values.

**Table 2:** Baseline Demographic and Clinical Characteristics of the Study Cohort.

Parameter	PR (*n* = 40)	PR+CS (*n* = 40)	*p* Value	Effect Size
Age (years)	52.1 ± 6.7	53.4 ± 6.4	0.377	−0.20
Symptom duration (months)	7.0 ± 1.9	6.8 ± 1.5	0.563	0.13
Baseline QuickDASH	48.5 ± 6.8	49.3 ± 7.2	0.623	−0.11
Baseline MHQ	61.5 ± 7.5	60.7 ± 6.5	0.623	0.11
Baseline VAS	6.5 ± 1.6	7.0 ± 1.4	0.141	−0.33
Sex, Female, *n* (%)	23 (57.5%)	25 (62.5%)	0.648	0.05
Diabetes mellitus, *n* (%)	9 (22.5%)	11 (27.5%)	0.606	0.06
Thumb involvement, *n* (%)	15 (37.5%)	16 (40.0%)	0.818	0.03
Green grade (2/3/4), *n*	16/14/10	14/13/13	0.755	0.08

Values are presented as mean ± standard deviation (SD) for continuous variables and as number (percentage) for categorical variables. Between-group comparisons were performed using the independent samples *t*-test for continuous variables and chi-square or Fisher’s exact test for categorical variables. Effect sizes were calculated using Cohen’s d for continuous variables and Cramér’s V for categorical variables. No statistically significant differences were observed between groups at baseline.

**Table 3:** Postoperative Outcomes and PASS Achievement at 3 Months.

Parameter	PR (*n* = 40)	PR+CS (*n* = 40)	*p* Value	Effect Size (d/V)
Month 3 QuickDASH	28.6 ± 8.0	25.1 ± 7.3	0.044 *	0.46
Month 3 MHQ	76.5 ± 8.4	81.2 ± 7.6	0.010 *	−0.59
Month 3 VAS	3.5 ± 1.7	2.8 ± 1.6	0.063	0.42
Δ QuickDASH	19.9 ± 3.2	24.2 ± 3.0	<0.001 *	−1.39
Δ MHQ	15.0 ± 3.3	20.5 ± 3.3	<0.001 *	−1.67
Δ VAS	3.0 ± 0.78	4.2 ± 0.72	<0.001 *	−1.59
PASS achieved, *n* (%)	22 (55.0%)	30 (75.0%)	0.061	0.21

Values are presented as mean ± standard deviation (SD) for continuous variables and as number (percentage) for categorical variables. Independent samples *t*-test was used for continuous variables; chi-square test for categorical variables. Effect sizes: Cohen’s d for continuous variables; Cramér’s V (V) for categorical variables. Δ denotes change from baseline to 3 months. PASS: patient acceptable symptom state. * *p* < 0.05.

## Data Availability

The data presented in this study are available on request from the corresponding author.

## Abbreviations

The following abbreviations are used in this manuscript:

MCID	Minimal Clinically Important Difference
SCB	Substantial Clinical Benefit
PASS	Patient Acceptable Symptom State
PR	Percutaneous Release
PR+CS	Percutaneous Release with Corticosteroid Injection
VAS	Visual Analog Scale
GROC	Global Rating of Change
MHQ	Michigan Hand Outcomes Questionnaire
QuickDASH	Quick Disabilities of the Arm, Shoulder and Hand
ROC	Receiver Operating Characteristic
AUC	Area Under the Curve
CI	Confidence Interval
PPV	Positive Predictive Value
NPV	Negative Predictive Value
PROM	Patient-Reported Outcome Measure
SD	Standard Deviation
IRB	Institutional Review Board
